# Comparison of Endoscopic Techniques in the Management of Type I Gastric Neuroendocrine Neoplasia: A Systematic Review

**DOI:** 10.1155/2021/6679397

**Published:** 2021-03-30

**Authors:** Francesco Panzuto, Ludovica Magi, Gianluca Esposito, Maria Rinzivillo, Bruno Annibale

**Affiliations:** ^1^Digestive Disease Unit, Sant'Andrea University Hospital, ENETS Center of Excellence, Rome, Italy; ^2^Department of Translational and Precision Medicine, Sapienza University of Rome, Italy; ^3^Department of Medical-Surgical Sciences and Translational Medicine, Sapienza University of Rome, Italy

## Abstract

**Background:**

Endoscopic resection is considered the treatment of choice for type I gastric neuroendocrine neoplasia (gNEN) given its indolent behaviour; however, the favoured endoscopic technique to remove these tumours is not well established.

**Aims:**

This systematic review is aimed at investigating the best endoscopic management for type I gNEN.

**Methods:**

PubMed Central/Medline and Scopus were systematically searched for records up to August 31, 2020.

**Results:**

After screening the 675 retrieved records, 6 studies were selected for the final analysis. The main endoscopic resection techniques described were endoscopic mucosal resection (EMR) and endoscopic submucosal dissection (ESD). Overall, 112 gNENs were removed by EMR and 77 by ESD. Both techniques showed similar results for complete and *en bloc* resection (97.4% and 98.7%; 92.3% and 96.3% with ESD and EMR, respectively). ESD was associated with a higher rate of complications than EMR (11.7% vs. 5.4%), but this difference was not statistically significant (*p* = 0.17). The rates of recurrence during follow-up were 18.2% and 11.5% for EMR and ESD, respectively.

**Conclusions:**

To date, there are no sufficient data showing superiority of a given endoscopic technique over others. Both ESD and EMR seem to be effective in the management of type I gNEN, with a relatively low rate of recurrence.

## 1. Introduction

Gastric neuroendocrine neoplasia (gNEN) is a heterogeneous group representing the most frequent gastroenteropancreatic NEN. Although gNENs account for less than 2% of gastric neoplasms, their incidence has been rising over the past 30 years due to the increasing use of upper endoscopy [1]. Concerning their different behaviours and prognoses, gNENs are clinically divided into three major types [2–4]. Type I gNENs are usually characterized by well-differentiated neoplasia with indolent behaviour and low metastatic potential; type II gNENs account for 5-10% of gNENs, with intermediate aggressiveness mostly associated with multiple endocrine neoplasia- (MEN-) 1 syndrome; and type III gNENs are rare and sporadic with aggressive behaviour, a high risk of metastasis, and poor long-term clinical outcomes.

Type I gNENs are the most frequent, accounting for 70-80% of all gNENs. They arise in the context of atrophic body gastritis (ABG) from enterochromaffin-like cells through a multistep process, passing from hyperplasia to dysplasia and then to NEN. Tumour progression is promoted by hypergastrinemia as a consequence of acid suppression and loss of parietal cells of the gastric body mucosa [5, 6]. Type I gNENs are diagnosed during endoscopies of the upper gastrointestinal tract usually carried out for anaemia or nonspecific symptoms (e.g., dyspepsia), corresponding to 0.6-2% of gastric polyp cases [1]. They are usually small and multifocal polyps appearing pale and yellowish with transparent blood vessels under white light endoscopy, while when using electronic chromoendoscopy, they present as regular mucosal and vascular patterns with central erosion and sometimes with a clear demarcation line [7]. However, a small proportion may also present as a normal-appearing mucosa (microcarcinoids) incidentally diagnosed on gastric biopsy [8]. Due to the good prognosis and low incidence of metastasis of gNENs, it is widely accepted that conservative treatment by endoscopic resection and surveillance is preferred over surgery [9, 10]. In general, endoscopic resection is well used for early stage neoplastic lesions of the gastrointestinal tract with a low risk of complications, mostly conservatively managed [11]. The main complications are bleeding and perforation. Bleeding may occur during the endoscopic procedure, such as intraprocedural bleeding, or later, until 30 days after resection, defined as postprocedural bleeding or delayed bleeding.

Beyond the tumour type, size is well recognized as one of the main prognostic factors in gNENs, affecting patient management and driving the best therapeutic choice [12]. European guidelines [11] recommend endoscopic submucosal dissection (ESD) as the treatment of choice for gastric superficial neoplastic lesions with a low risk of lymph node metastasis, and it is considered the best technique to achieve complete *en bloc* resection for early gastric cancer even for Japanese guidelines [13]. Endoscopic mucosal resection (EMR) could be an option for lesions smaller than 10-15 mm with a low risk of advanced histology [11]. EMR seems to be safer, but ESD has the advantage of *en bloc* resection for a complete histological evaluation. Since the favoured endoscopic technique to remove gastric NENs is not well established, this systematic review aimed at investigating is the best endoscopic technique to manage patients with type I gNENs.

## 2. Materials and Methods

### 2.1. Inclusion Criteria

Studies evaluating endoscopic resection in type I gNENs were included, regardless of the publication date, study design (prospective or retrospective), or sample size. Only studies published in the English language were selected. Case reports were excluded, as well as those studies evaluating gNENs other than type I.

### 2.2. Search Strategy

Medline searches through PubMed and Scopus databases were conducted in August 2020 using the following search query: (gastric carcinoid OR gastric neuroendocrine tumour OR gastric neuroendocrine neoplasia) AND (endoscopic resection OR mucosal resection OR submucosal dissection OR polypectomy), according to the Preferred Reporting Items for Systematic Review and Meta-Analyses (PRISMA) statement [14] (suppl. file 1). The protocol was registered on the Prospero platform (ID no. CRD42020203361). The initial screening included evaluations of titles and abstracts to check whether inclusion criteria were satisfied. A full-text analysis was performed for those studies that were included in the following final evaluation. The references of all selected papers were further reviewed to identify additional relevant articles, which were analysed by reading titles, abstracts, and eventually full texts according to the inclusion criteria.

### 2.3. Data Extraction, Outcome Measures, and Statistical Analysis

Two reviewers (MR and LM) independently performed the search, study selection, and data extraction. In the case of disagreement between the two reviewers, a third blinded reviewer was involved (FP). The following features were recorded by each selected study, which was included in the final analysis: number of included patients, number of resected tumours, median tumour size, and type of endoscopic procedure performed to remove tumours. The primary outcomes were the proportion of radically resected lesions with tumour-free margins (R0 resection), the number and types of resection-related adverse events, and disease recurrence-free survival after resection. Data extracted from the included studies were collected according to the endoscopic technique reported, and a comparison between the outcomes was carried out using Fisher's exact test. A *p* value of <0.05 was considered statistically significant.

## 3. Results

**Figure 1:**
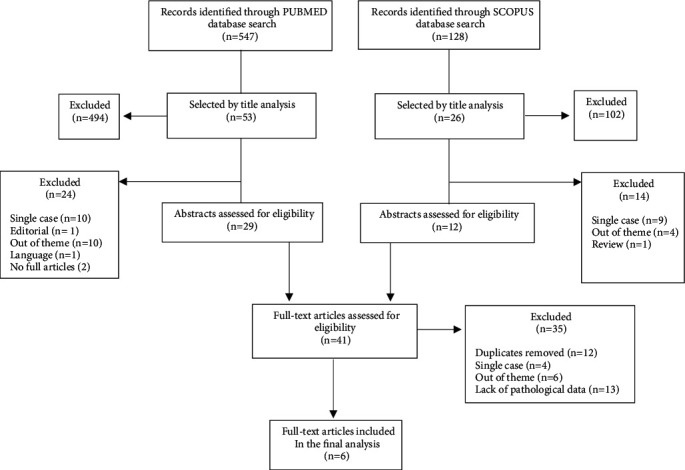
Search strategy.

The results yielded by the initial algorithm and the successive steps of the selection process are described in Figure 1. From the 675 retrieved studies, 6 [15–20] were finally considered eligible, with 100% agreement between the two reviewers. The most frequent reason for exclusion was the lack of specification of gNEN type.

The characteristics of the selected studies are summarized in Table 1. Four studies included only patients with a histological diagnosis of type I gNEN, while one study reported patients with all types of gNENs and one with a mixed population including gastric and intestinal NENs; specific data on type I gNEN were extracted from these studies and included in the final analysis. Overall, the most frequent aim of these studies was to investigate the efficacy of endoscopic resection. Two studies [16, 19] focused on long-term outcomes, and the recurrence rate was the main endpoint. Only one single study directly compared two resection techniques, investigating the clinical outcomes of patients treated with EMR vs. ESD [15].

### 3.1. Efficacy of Endoscopic Resection

After pooling the data extracted from the selected records included in the final analysis, 112 gNENs were found to be removed by EMR and 77 by ESD (Table 2). The probability of achieving complete resection (R0) was not significantly different between the two groups: complete resection was reported in 104/112 (92.3%) and 75/77 (97.4%) lesions resected by EMR and ESD, respectively. Additionally, for *en bloc* resection, both techniques showed similar results. Since one study did not report data on *en bloc* resection with EMR, data on this outcome were available in 81 out of 112 patients treated by EMR. Overall, complete tumour removal was obtained after *en bloc* resection in 78/81 (96.3%) and 76/77 (98.7%) by EMR and ESD, respectively.

Among the selected studies, only one [19] performed in 33 patients used either a snare or forceps. In that study, a 100% complete resection rate was reported; however, the median diameter of lesions resected in that study was 5 mm, and a high recurrence rate was observed during follow-up.

### 3.2. Resection-Related Complications

The overall rate of complications was similar for both EMR and ESD (5.4% vs. 11.7%; *p* = 0.17), occurring in 7 and 9 patients, respectively (Table 2). Specifically, intraprocedural bleeding occurred in 3 (6.2%) and 6 patients (15.4%) treated with EMR and ESD, respectively, whereas postprocedural bleeding was reported in 2 cases for both endoscopic techniques (4.2% and 5.1% for EMR and ESD, respectively). The overall bleeding rate was higher in patients who underwent ESD (10.4%) than in those who were treated by EMR (4.5%); however, this difference was not statistically significant (*p* = 0.14). Finally, one instance of perforation was reported either with EMR or with ESD resection. No complications were reported in the single study in which forceps and snare resection were used [19].

An additional finding recorded by three of the included studies [15, 17, 18] was the procedure length; however, a pooled analysis was not feasible for this parameter, owing to the paucity and heterogeneity of the available data. Overall, a mean procedure duration of 21.4 minutes (range 10-45 minutes) was recorded for the two studies evaluating ESD resection [17, 18], whereas the single comparative study [15] showed a significantly longer procedure time with ESD (26.1 ± 10.5 minutes) than with EMR (9.5 ± 3.6 minutes), with a *p* value < 0.001.

### 3.3. Recurrence after Endoscopic Resection

Data on the risk of recurrence were reported in four studies. The follow-up period of patients after resection ranged from 24.4 months to 7 years. Only one study [16] reported data on recurrence after EMR, with a rate of 18.2% during a median follow-up of 7 years. After ESD, two studies [17, 18] reported a rate of recurrence of 11.5%; however, the median follow-up was less than 30 months for both studies. The rate of recurrence was significantly higher (63.6%) when resection was performed by forceps or a snare [19], with a significant difference compared to resection performed by EMR and/or ESD (*p* < 0.0001).

## 4. Discussion

It is well known that the achievement of complete resection is crucial to establish correct management in type I gNENs. Even though they are considered indolent diseases, the risk of metastasis and lymph node involvement is not zero, particularly for lesions > 10 mm in diameter [12, 21, 22].

This systematic review shows that both EMR and ESD are effective techniques to achieve complete resection in type I gNENs. Among the studies included in the final analysis, only one [15] directly compared both endoscopic techniques, showing the superiority of ESD over EMR for achieving complete resection (94.9% vs. 83.3%). However, after pooling data from the selected studies, both techniques seem to have similar efficacy, with no clear superiority in terms of the complete resection rate for ESD compared with EMR (97.4% vs. 92.3%, respectively). Based on these data, it may be assumed that both endoscopic techniques are effective in managing patients with type I gNENs, given their excellent, similar rates of complete resection.

As far as snare polypectomy is concerned, although it is widely used in endoscopy for polyps less than 10 mm, it seems not to be effective in the particular setting of gNENs with major involvement of the submucosa layer [23]. Data on snare polypectomy in gNENs are even scarcer, owing to the presence of one single study evaluating 33 patients among those selected for the final analysis in the present review [19]. In that study, complete resection was reported in all patients; however, a high rate of tumour recurrence was observed, suggesting that simple forceps/snare polypectomy is not adequate to achieve complete long-term resection of gNENs.

Endoscopic full-thickness resection (EFTR) has also been proposed as an effective tool to remove deep gastric submucosal tumours [24], however without available data focusing on gNENs and a few case reports on rectal NENs [25, 26].

When the complication rate was investigated, no significant advantage was observed for a given endoscopic technique. Both ESD and EMR were associated with low, similar risks for immediate and postprocedural bleeding and perforation. Conversely, no complications were recorded with snare polypectomy, which, as expected, is well known as a safer procedure. As an additional finding, even if a pooled analysis was not performed, this systematic review highlights that the procedure time with ESD may be longer than that with EMR [15], in accordance with the current literature [27].

Regarding the risk of recurrence during follow-up, significantly variable findings were observed according to the specific endoscopic technique, and ESD showed a lower risk than other techniques. In general, although gNENs are considered indolent neoplasms, recurrence frequently occurs during follow-up, even many years after the initial diagnosis, [28] suggesting that long-term endoscopic follow-up is advised for gNEN patients. A significant risk of recurrence was also reported by a recent study, published after this systematic search was performed, including 12 patients with gNEN treated by EMR. In this group of patients, around half of cases required further EMR during endoscopic surveillance after initial tumour resection [29]. Unfortunately, analysis of the real risk of recurrence according to a specific endoscopic technique is difficult to perform. Only four studies reported the risk of recurrence, moreover with an extremely heterogeneous follow-up period. Furthermore, an additional point needs to be taken into account when analysing this issue; that is, the difficulty of establishing whether tumour recurrence is related to local residual disease or to the onset of new tumour lesions as a continuous stimulus by hypergastrinemia, which occurs in patients with ABG.

Endoscopic ultrasonography might be an additional useful tool for investigating tumour depth and to rule out the presence of lymph node metastases which, although rare, may occur particularly in tumours > 10 mm [12]. Unfortunately, only a few of the evaluated studies [15, 17, 18] included EUS in the diagnostic evaluation of the tumours which, however, were small gNENs < 10 mm. Thus, addressing the issue regarding the role of EUS in the selected group of high-risk gNENs > 10 mm is not feasible.

Evaluating data on the real efficacy and safety of the available endoscopic techniques is difficult, owing to the lack of comparative studies, the retrospective design of most of the evaluated studies, and the low number of patients included. The present review attempts to pool and analyse these data with the robust methodology of a systematic approach, focusing on the possible differences among the reported techniques in terms of efficacy and safety. However, this work has some limits: (i) the median diameter of resected lesions was relatively low, usually less than 10 mm, which might explain the similarity in terms of clinical outcomes for EMR and ESD, especially for those tumours removed by *en bloc* resection; (ii) most of the evaluated studies included a low number of patients; (iii) a subanalysis among the different techniques of EMR (e.g., snare resection, cap-assisted) was not feasible, and this might play a role when analysing the proportion of complete resection; and (iv) only one study compared ESD and EMR.

## 5. Conclusion

The optimal endoscopic management strategy for patients with type I gNEN remains a challenge for physicians dealing with this rare kind of tumour. The present systematic review highlights the lack of solid data able to provide superiority of a given endoscopic technique over others. Both EMR and ESD are effective techniques for achieving complete resection; however, EMR seems to be associated with a low rate of complications. No conclusion may be drawn for forceps/snare polypectomy given the paucity of scientific data on this issue. However, the extremely high proportion of disease recurrence during follow-up seems to discourage this technique for type I gNEN removal.

Prospective, comparative large studies including a homogeneous population of type I gNENs evaluating the different techniques are mandatory to understand the best therapeutic option for these patients.

## Figures and Tables

**Table 1:** Characteristics of the selected studies.

First author [ref]	Study design	No. of patients	ER^a^ technique	Diameter of lesion	Complete resection	Complications	Recurrence
Kim [15]	Multi-center retrospective cohort study	62	EMR^b^ or ESD^c^	Mean: 7.6 mm	EMR^b^: 83.3% ESD^c^: 94.9%	EMR^b^: 10.4% ESD^c^: 23.1%	NR
Uygun [16]	Single-center prospective cohort study	22	EMR^b^	5-20 mm	100%	4.4%	18.2%
Li [17]	Single-center retrospective cohort study	11	ESD^c^	5-8 mm	100%	0%	0%
Chen [18]	Single-center retrospective cohort study	15	ESD^c^	2-30 mm	100%	0%	13.3%
Merola [19]	Single-center prospective cohort study	33	Forceps or snare	2-20 mm	100%	0%	63.6%
Hopper [20]	Multicenter prospective cohort study	8	EMR^b^	0,6-10 mm	100%	0%	NR

**Table 2:** Results after pooling data extracted from the selected records.

Records included in the final analysis	ER^a^ technique	No. of patients	No. of lesions	Complete resection	En bloc resection	Complications	Recurrence
Kim [15] Li [17] Chen [18]	ESD^b^	65	77	75/77 (97.4%)	76/77 (98.7%)	9/77 (11.7%)	3/26 (11.5%)
Kim [15] Uygun [16] Hopper [20]	EMR^c^	55	112	104/112 (92.3%)	78/81 (96.3%)	6/112 (5.4%)	4/22 (18.2%)
*p* value				0.2	0.62	0.17	0.68
